# Comparative Assessment of the WNT/β-Catenin Pathway, CacyBP/SIP, and the Immunoproteasome Subunit LMP7 in Various Histological Types of Renal Cell Carcinoma

**DOI:** 10.3389/fonc.2020.566637

**Published:** 2020-11-20

**Authors:** Żaneta Piotrowska, Michał Niezgoda, Grzegorz Młynarczyk, Magdalena Acewicz, Irena Kasacka

**Affiliations:** ^1^ Department of Histology and Cytophysiology, Medical University of Białystok, Białystok, Poland; ^2^ Department of Urology, Medical University of Białystok, Białystok, Poland

**Keywords:** WNT/β-catenin pathway, CacyBP/SIP, LMP7 immunosubunit, renal cell carcinoma, human

## Abstract

**Objective:**

The Wnt/ß-catenin pathway plays an important role in pathogenesis of variety cancers. Most studies on changes in WNT/β-catenin pathway in renal cell carcinoma (RCC) apply only to clear cell RCC, while there are no comparative assessments of this signaling pathway in various histological types of renal tumors in the available literature. Additionally, considering the close relationship between WNT/β-catenin signaling, CacyBP/SIP and proteasomal activity, it seemed worth comparing WNT/β-catenin pathway, CacyBP/SIP and LMP7 immunoproteasome subunit in human samples of clear cell, papillary, and chromophobe RCC.

**Methods:**

Tests were performed on sections of three types of kidney tumors together with surrounding unchanged tissue fragments collected from 50 patients. Samples were divided into three groups depending on the histological type of cancer: clear cell, papillary and chromophobe RCC. Immunohistochemistry and PCR methods were used to identify WNT10A, Fzd5, β-catenin, GSK-3ß, CacyBP/SIP, LMP7, and gene expression.

**Results:**

Immunoreactivity and expression of WNT10A, Fzd5, β-catenin, GSK-3ß, CacyBP/SIP, LMP7 in clear cell RCC was markedly increased compared to non-cancerous kidney tissue. In papillary RCC, immunoreactivity and expression of WNT/β-catenin pathway, CacyBP/SIP, LMP7 was also increased compared to non-malignant kidneys, but it was less pronounced than in clear cell RCC. The least substantial increase in immunoreactivity and expression of WNT/β-catenin pathway, CacyBP/SIP, LMP7 was found in chromophobe RCC, compared to other RCC histological subtypes studied.

**Conclusions:**

Study results suggest an important role of WNT/β-catenin pathway, CacyBP/SIP and LMP7 in RCC carcinogenesis, and may indicate new aspects of pathomechanisms leading to differences in the biology of clear cell, papillary and chromophobe RCC.

## Introduction

Renal cell carcinoma (RCC) accounts for approximately 2% to 3% of all human cancers (1). The incidence of RCC is far higher in men who represent around two-thirds of all cases (1). Approximately 40% of RCC patients die of the disease, which makes it the third most deadly cancer among urological tumors (1). 25% to 30% of RCC patients are diagnosed with advanced metastatic disease and a further 30% to 50% of individuals with primary RCC develop metastases (2). The survival rate of patients with metastases is around 48% in the first year and only 9% five years after diagnosis (2). The most common histological subtypes of RCC are clear cell RCC (66–75% of cases), papillary RCC (10–15% of cases) and chromophobe RCC (5% cases) (1, 3). Clear cell RCC is associated with the highest invasiveness and shortest cancer-specific survival rate, while chromophobe RCC is related to the lowest aggressiveness and best prognosis compared to other RCC histological subtypes (1, 3, 4).

The Wnt/ß-catenin signaling pathway plays an important role in the process of carcinogenesis (5). It includes specific Frizzled membrane receptors (Fzd), endogenous agonists—WNT proteins as well as ß-catenin—the main effector of the pathway (5–7). ß-catenin is a bifunctional protein since it determines many aspects of cell physiology as a transcription factor, i.e. proliferation, differentiation, survival and migration, while as a membrane-bound protein, it coordinates intercellular adhesion and is responsible for maintaining tissue architecture (5–7). In the absence of binding of WNT ligands to Fzd receptors, intracellular ß-catenin levels are kept very low due to continuous degradation. The level of cytoplasmic ß-catenin is regulated by a multi-protein complex, called the ß-catenin destruction complex, consisting of axin, adenomatous polyposis coli (APC), casein kinase 1 (CK1) and glycogen synthase kinase 3 ß (GSK-3ß). In this complex, ß-catenin is phosphorylated by a dual-kinase mechanism involving CKI and GSK-3ß, and then phosphorylated ß-catenin is ubiquitinated and is directed to proteasomal degradation. Following the attachment of the WNT ligand and activation of Fzd receptors, destruction of ß-catenin is inhibited and the peptide accumulates in the cell. Then ß-catenin is transferred to the nucleus, where it regulates the expression of target WNT genes (5–7).

Increased activity of the WNT/β-catenin pathway has been found in various types of cancer such as colorectal cancer, hepatocellular carcinomas, melanoma, lung cancer, leukemia, bladder cancer, breast cancer, ovarian cancer and cervical cancer (8, 9). It has been demonstrated that the WNT/β-catenin pathway is involved in the pathogenesis and progression of RCC, tumor angiogenesis and invasive behavior of transformed cells (6). Several clinical and experimental studies have shown increased expression of WNT proteins and Fzd receptors, as well as increased content of β-catenin in RCC (6–12). Literature data also indicate shorter survival for RCC patients who have been found to have increased expression of WNT proteins or accumulated β-catenin in tumor tissue (9–11). Therefore, hyperactivation of WNT/β-catenin signaling may be of clinical significance in RCC (9–11). Genetic silencing of the WNT/β-catenin pathway in RCC cell lines has been demonstrated to result in inhibition of proliferation and migration, induction of apoptosis and increase of cancer cells sensitivity to chemotherapy (11).

Some recent evidence suggests that GSK-3ß, a key kinase of the aforementioned β-catenin destruction complex, may also be a promising therapeutic target in RCC (13). Increased GSK-3ß content has been found in human RCC samples and cultured RCC cell lines (13–15). Genetic or pharmacological inhibition of GSK-3ß contributes to cell cycle arrest and reduces viability of RCC cell lines (13–15).

An alternative pathway of β-catenin degradation with participation of the CacyBP/SIP protein (Calcyclin-binding protein/Siah-1–interacting protein) has recently been discovered (16). CacyBP/SIP is a multi-domain protein that interacts with a wide range of intracellular molecules, including ubiquitin ligases components Siah-1 (seven in absentia homolog-1) and Skp1 (S-phase kinase associated protein 1). CacyBP/SIP attaches to Siah-1 and Skp1, stabilizes the ubiquitin ligase complex and promotes the degradation of non-phosphorylated β-catenin (16). Independent scientific centers have indicated the importance of CacyBP/SIP in the process of cancer formation. Abnormal CacyBP/SIP levels have been found in pancreatic cancer, gastric cancer, colorectal cancer, osteogenic sarcoma, melanoma, kidney cancer, breast cancer, brain cancer (16).

It has been shown that CacyBP/SIP can act as a tumor suppressor or an oncogen depending on the type of cancer (16–18). Decreased CacyBP/SIP expression has been found in samples taken from patients with RCC as well as in several RCC lines (19). Forced overexpression of CacyBP/SIP in RCC cell lines has been shown to reduce the proliferative potential of cancer cells *in vitro* and their carcinogenicity when injected into mice (19).

The body’s immune system constantly attacks and destroys cancer cells that form. Disturbances in antigen presentation on cell surfaces have been found in various types of cancers, which may be a mechanism for avoiding recognition and destruction by the immune system (20–22). The crucial role in antigen processing is played by immunoproteasomes involved in the production of antigenic peptides and the major histocompatibility complex class I (MHC I), which is responsible for presentation of antigens to immunologically competent cells (20–22). Studies on patients with RCC have shown reduced expression of both immunoproteasomes and MHC I in malignant kidney samples. These findings indicate that in RCC cells, tumor antigen presentation may be inhibited in order to avoid an anti-tumor immune response (20–22).

Most studies on changes in the WNT/β-catenin pathway in RCC focus only on clear cell RCC, while there is no published literature on the comparative assessment of this signaling pathway in different histological types of RCC (6–12). Additionally, considering the close relationship between WNT/β-catenin signaling, CacyBP/SIP and proteasomal activity, it seemed worth comparing the WNT/β-catenin pathway, CacyBP/SIP and LMP7 immunoproteasome subunit in human samples of clear cell, papillary and chromophobe RCC.

The aim of this study is immunohistochemical evaluation and comparison of the expression of the WNT10A, Fzd5, β-catenin, GSK-3ß, CacyBP/SIP, and LMP7 genes in human kidney cancer tissue in different histological types of RCC.

## Material and Methods

### Sample Collection

The research was conducted on postoperative material collected from fifty patients of Department of Urology, Medical University of Bialystok, operated on for kidney cancer. The study protocol was approved by the Bioethics Committee, Medical University of Bialystok (R-I-002/282/2019) and prior written informed consent was obtained from each subject.

The research material were fragments of RCC lesions obtained during radical or partial nephrectomy. The comparative material were fragments of surrounding unchanged kidney tissue (margins). All RCC lesions were at the same grade G2, except for chromophobe RCC in which grading system is not applicable. Obtained malignant and non-cancerous kidney tissues were immediately fixed in Bouin’s solution and routinely embedded in paraffin or placed in RNA-later solution (AM7024 Thermo Fischer) and stored in −80°C.

The subjects were divided into 3 groups, depending on the histological type of cancer: clear cell RCC (35 cases), papillary RCC (12 cases) and chromophobe RCC (3 cases).

Renal paraffin blocks were cut into 4 µm section thickness and then stained with hematoxylin-eosin for general histological examination and processed by immunohistochemistry to detect WNT10A, Fzd5, β-catenin, GSK-3ß, CacyBP/SIP, and LMP7. Material stored in RNA-later solution was processed by real-time PCR to evaluate the expression of genes coding WNT10A, Fzd5, β-catenin, GSK-3ß, CacyBP/SIP, and LMP7.

### Immunohistochemistry

In the immunohistochemical study, the EnVision method was used according to Herman and Elfont (23). Immunohistochemistry was performed, using an REAL™ EnVision™ Detection System, Peroxidase/DAB, Rabbit/Mouse detection kit (K5007; DakoCytomation; Glostrup, Denmark). Immunostaining was performed by the following protocol: paraffin-embedded sections were deparaffinized and hydrated in pure alcohols. For antigen retrieval, the sections were subjected to pretreatment in a pressure chamber heated for 1 min at 21 psi (one pound force per square inch (1 psi) equates to 6.895 kPa, the conversion factor has been provided by the United Kingdom National Physical Laboratory) at 125°C, using Target Retrieval Solution Citrate pH=6.0 S 2369 (DakoCytomation; Glostrup, Denmark) for WNT10A, Fzd5, β-catenin, CacyBP/SIP and LMP7 and Target Retrieval Solution pH = 9.0 for GSK-3ß. After cooling down to room temperature, the sections were incubated with Peroxidase Blocking Reagent S 2001 (DakoCytomation; Glostrup, Denmark) for 10 min to block endogenous peroxidase activity. Subsequently sections were incubated with primary antibody for WNT10A (Rabbit polyclonal to Wnt10a ab106522 Abcam), Fzd5 (Rabbit polyclonal to Frizzled 5 ab115204 Abcam), β-catenin (Recombinant Anti-beta Catenin antibody (E247) ab32572 purchased from Abcam), GSK-3ß (Rabbit monoclonal (EPR933Y) to GSK3 (alpha + beta) (phospho Y216 + Y279) ab68476 Abcam), CacyBP/SIP (Rabbit polyclonal to CacyBP ab190950 Abcam) and LMP7 (mouse monoclonal antibody to LMP7, PW8845, purchased from Biomol). All antibodies were previously diluted in Antibody Diluent Background Reducing (S 3022 DakoCytomation; Glostrup, Denmark) in relation 1:1000 for WNT10A antibody, 1:150 for Fzd5 antibody, 1:2000 for β-catenin antibody, 1:100 for GSK-3ß antibody, 1:600 for CacyBP/SIP antibody and 1:5000 for LMP7 antibody. Sections with WNT10A-, Fzd5-, β-catenin-, GSK-3ß-, CacyBP/SIP-, and LMP7-antibody were incubated overnight at 4°C (incubation performed in a humidified chamber). The procedure was followed by incubation with secondary antibody (conjugated to horseradish peroxidase-labeled polymer). The bound antibodies were visualized by 1-min incubation with liquid 3,3′-diaminobenzidine substrate chromogen. The sections were finally counterstained in hematoxylin QS (H-3404, Vector Laboratories; Burlingame, CA), mounted, covered and evaluated under a light microscope. Appropriate washing with Wash Buffer (S 3006 DakoCytomation; Glostrup, Denmark) was performed between each step.

Specificity tests performed for WNT10A-, Fzd5-, β-catenin-, GSK-3ß-, CacyBP/SIP, and LMP7 included a negative control in which the antibodies were replaced by normal rabbit serum (Vector Laboratories; Burlingame, CA) with appropriate dilution. All these controls were negative.

Histological preparations were visually analyzed using an Olympus BX43 light microscope (Olympus 114 Corp., Tokyo, Japan) with an Olympus DP12 digital camera (Olympus 114 Corp., Tokyo, Japan) and documented.

### Quantitative Analysis

Twelve sections of malignant lesion and twelve section of non-cancerous kidney tissue were examined from each subject (two section for each WNT10A-, Fzd5-, β-catenin-, GSK-3ß-, CacyBP/SIP- and LMP7-immunostaining). Five randomly selected microscopic fields (each field 0.785 mm^2^, 200× magnification (20× lens and 10× eyepiece)) from each kidney section were documented using an Olympus DP12 microscope camera. Each obtained digital image of the kidney section was morphometric evaluated using NIS Elements AR 3.10 Nikon software for microscopic image analysis.

The intensity of the immunohistochemical reaction for all the antibodies used in the study was measured on each image analyzed and determined using a gray scale level 0 to 255, where the value of the completely white or bright pixel is 0, while the completely black pixel is 255.

### Real-Time PCR

Samples of kidney cancer and non-malignant renal tissue (1 cm^3^) were taken from each patient and placed in an RNA-later solution. Total RNA was isolated using NucleoSpin^®^ RNA Isolation Kit (Machery-Nagel). Quantification and quality control of total RNA was determined using a spectrophotometer - NanoDrop 2000 (ThermoScientific). An aliquot of 1 µg of total RNA was reverse transcribed into cDNA using iScript™ Advanced cDNA Synthesis Kit for RT-qPCR (BIO-RAD). Synthesis of cDNA was performed in a final volume of 20 μl using an Thermal Cycler (Model SureCycler 8800, Aligent Technologies). For reverse transcription, the mixtures were incubated at 46°C for 20 min, then heated to 95°C for 1 min and finally cooled quickly at 4°C. Quantitative real-time PCR reactions were performed using Stratagene Mx3005P (Aligent Technologies) with the SsoAdvanced™ Universal SYBER^®^ Green Supermix (BIO-RAD). Specific primers for WNT10A (*WNT10A*), Fzd5 (*FZD5*), β-catenin (*CTNNB1*), GSK-3ß (*GSK3B*), CacyBP/SIP (*CACY BP*), LMP7 (*PSMB8*), and GAPDH (*GAPDH*) were designed by BIO-RAD Company. The housekeeping gene GAPDH (*GAPDH*) was used as a reference gene for quantification. To determine the amounts of levels of test genes expression, standard curves were constructed for each gene separately with serially diluted PCR products. PCR products were obtained by cDNA amplification using specific primers as follows: *WNT10A* (qHsaCED0044646, BIO-RAD), *FZD5* (qHsaCJD0034589, BIO-RAD), *CTNNB1* (qHsaCED0046518, BIO-RAD), *GSK3B* (qHsaCED0057061, BIO-RAD), *CACY BP* (qHsaCED0043669, BIO-RAD), *PSMB8* (qHsaCED0037294, BIO-RAD), and *GAPDH* (qHsaCED0038674, BIO-RAD). qRT-PCR was carried out in a doublet in a final volume of 20 μl under the following conditions: 2 min polymerase activation at 95°C, 5 s denaturation at 95°C, 30 s annealing at 60°C for 35 cycles. PCR reactions were checked, including no-RT-controls, omitting of templates, and melting curve to ensure only one product was amplified. The relative quantification of gene expression was determined by comparing Ct values using the ΔΔCt method. All results were normalized to GAPDH.

### Statistical Analysis

All data were analyzed for statistical significance using the Statistica version 12.0 computer software package. The mean values were computed automatically; significant differences were determined by one-way ANOVA test; p < 0.05 was considered significant.

## Results

Baseline characteristics of patients with RCC, including the proportion of men and women, mean values of age, minimal and maximal value of age are presented in **Table 1**. Men consisted almost two-thirds of studied RCC cases. Patients with RCC belonged to middle-age group, most of the cases concerned individuals in the fifth or sixth decade of life (**Table 1**).

**Table 1:** Characteristic of patients with clear cell, papillary and chromophobe RCC.

Group of patients	Proportion of men	Proportion of women	Age [years]		
			Mean	Min	Max
Overall	32/50	18/50	66.2 ± 1.30	40	87
Clear cell RCC	20/35	15/35	67.0 ± 1.32	43	87
Papillary RCC	10/12	2/12	62.1 ± 6.10	40	82
Chromophobe RCC	2/3	1/3	59.3 ± 2.03	56	63

### Immunohistochemistry

**Figure 1:**
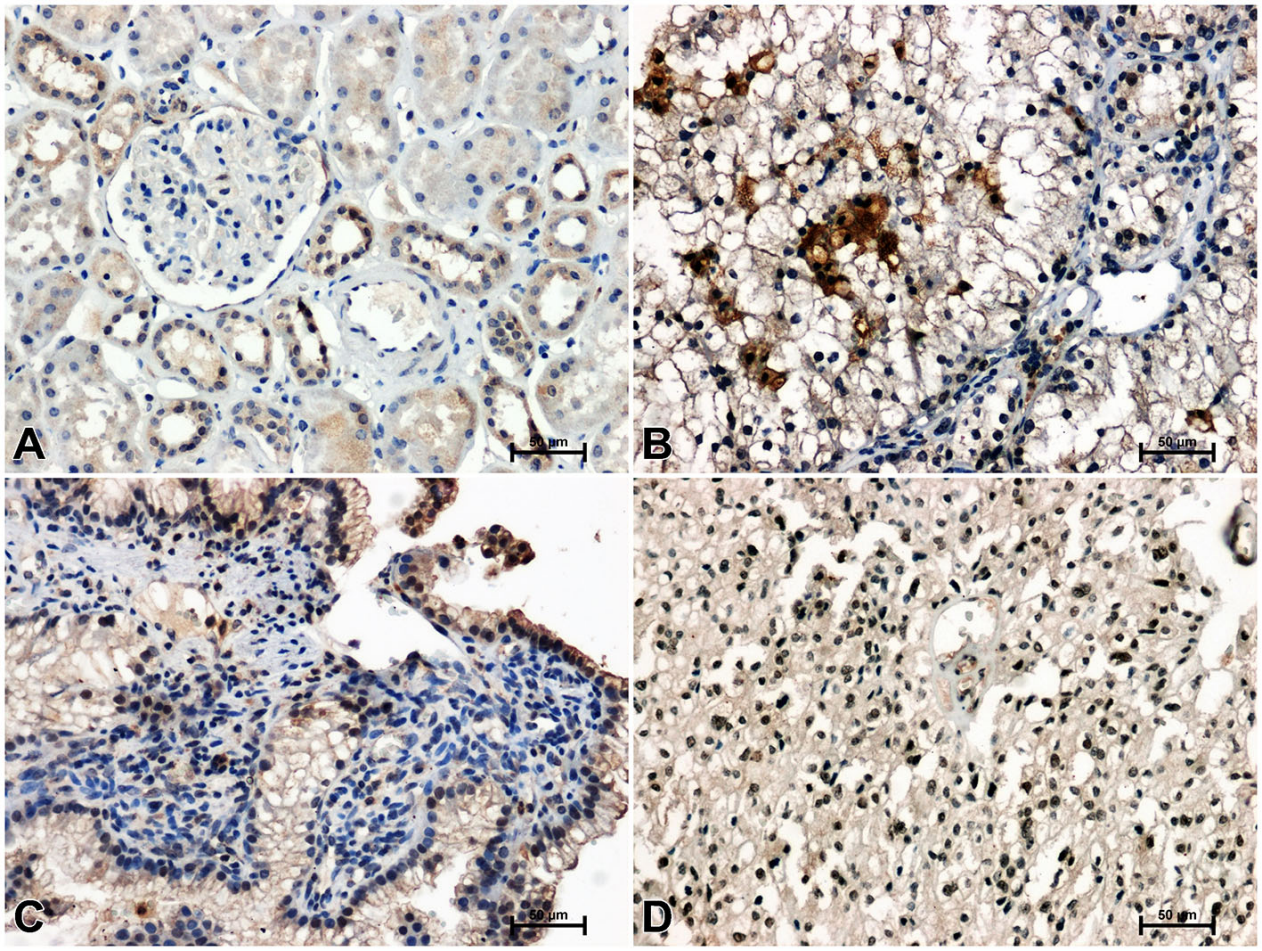
Immunoidentification of WNT10A in non-malignant kidney **(A)** and clear cell RCC **(B)**, papillary RCC **(C)**, chromophobe RCC **(D)**.

A positive immunohistochemical reaction for WNT10A, Fzd5, β-catenin, GSK-3ß, CacyBP/SIP, and LMP7 was observed in all studied kidneys tissues, although the intensity of immunoreaction varied between non-malignant kidney and RCC tissue samples, depending on the histological type of the tumor (**Figures 1** **–** **6**).

**Figure 2:**
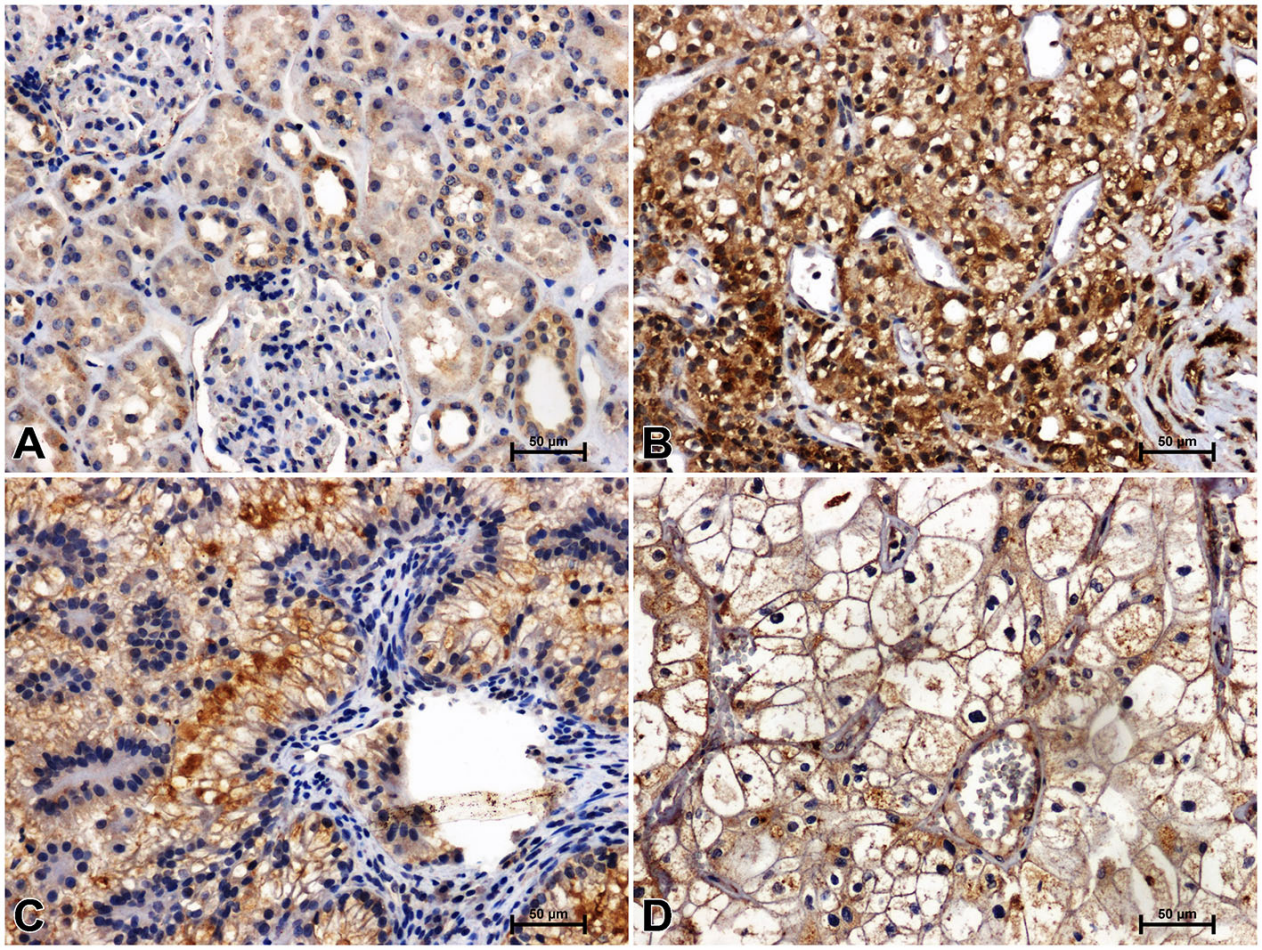
Immunodetection of Fzd5 in non-malignant kidney **(A)** and clear cell RCC **(B)**, papillary RCC **(C)**, chromophobe RCC **(D)**.

**Figure 3:**
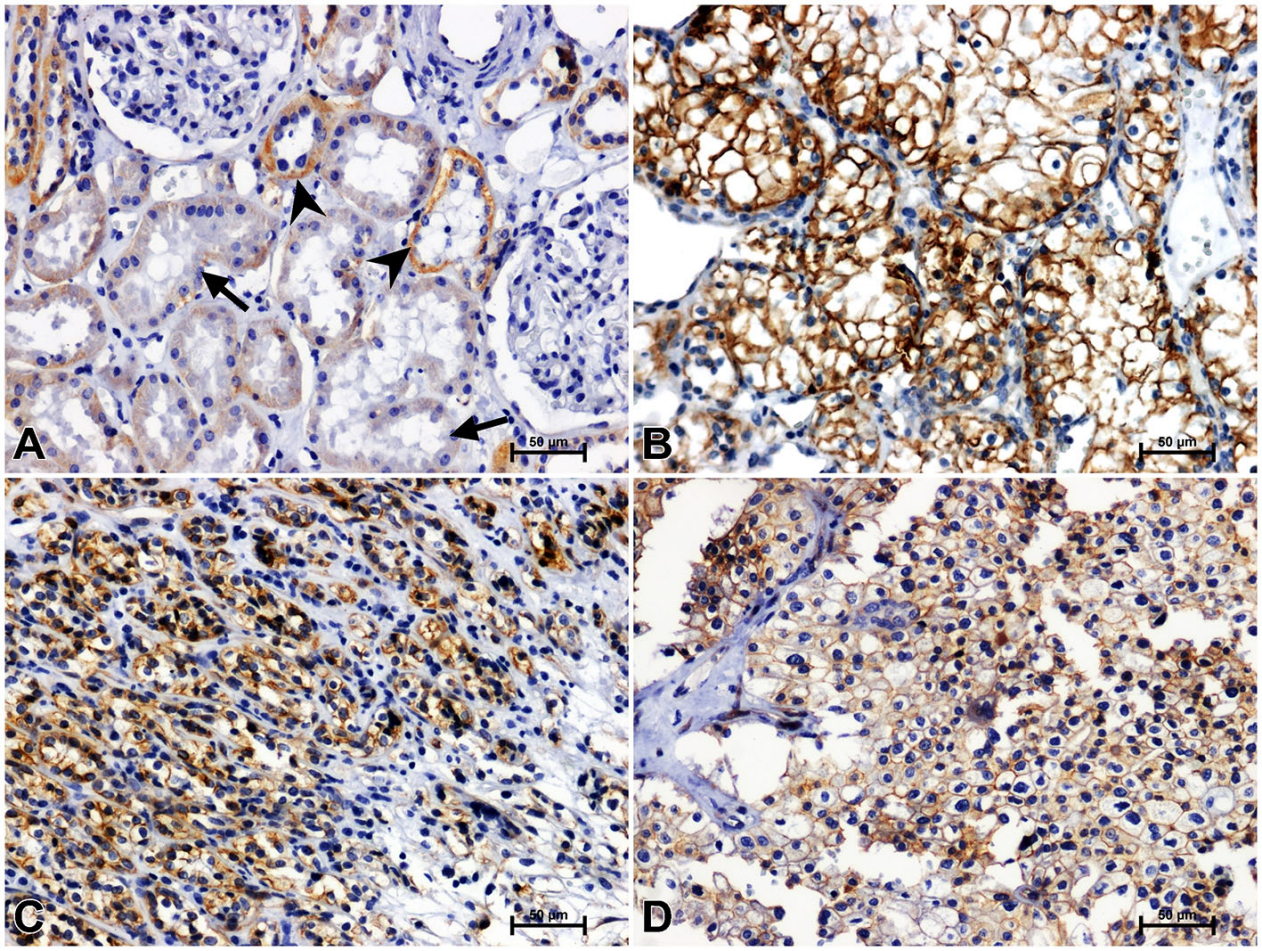
Positive β-catenin immunostaining in distal (arrowheads) and proximal (arrows) tubules of non-malignant kidney **(A)**. Results of β-catenin immunostainig in clear cell RCC **(B)**, papillary RCC **(C)**, chromophobe RCC **(D)**.

**Figure 4:**
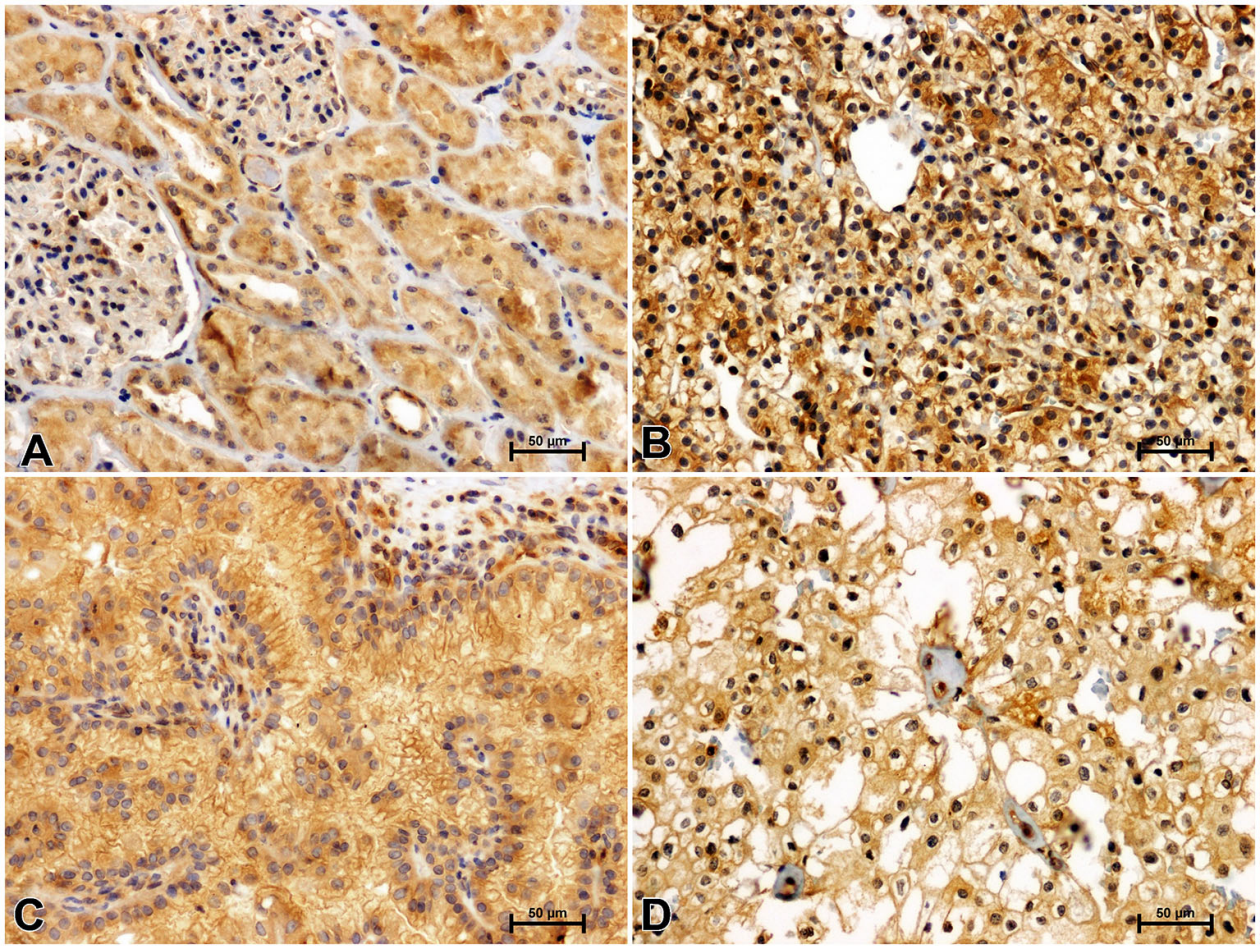
Immunolabeling of GSK-3ß in non-malignant kidney **(A)** and clear cell RCC **(B)**, papillary RCC **(C)**, chromophobe RCC **(D)**.

**Figure 5:**
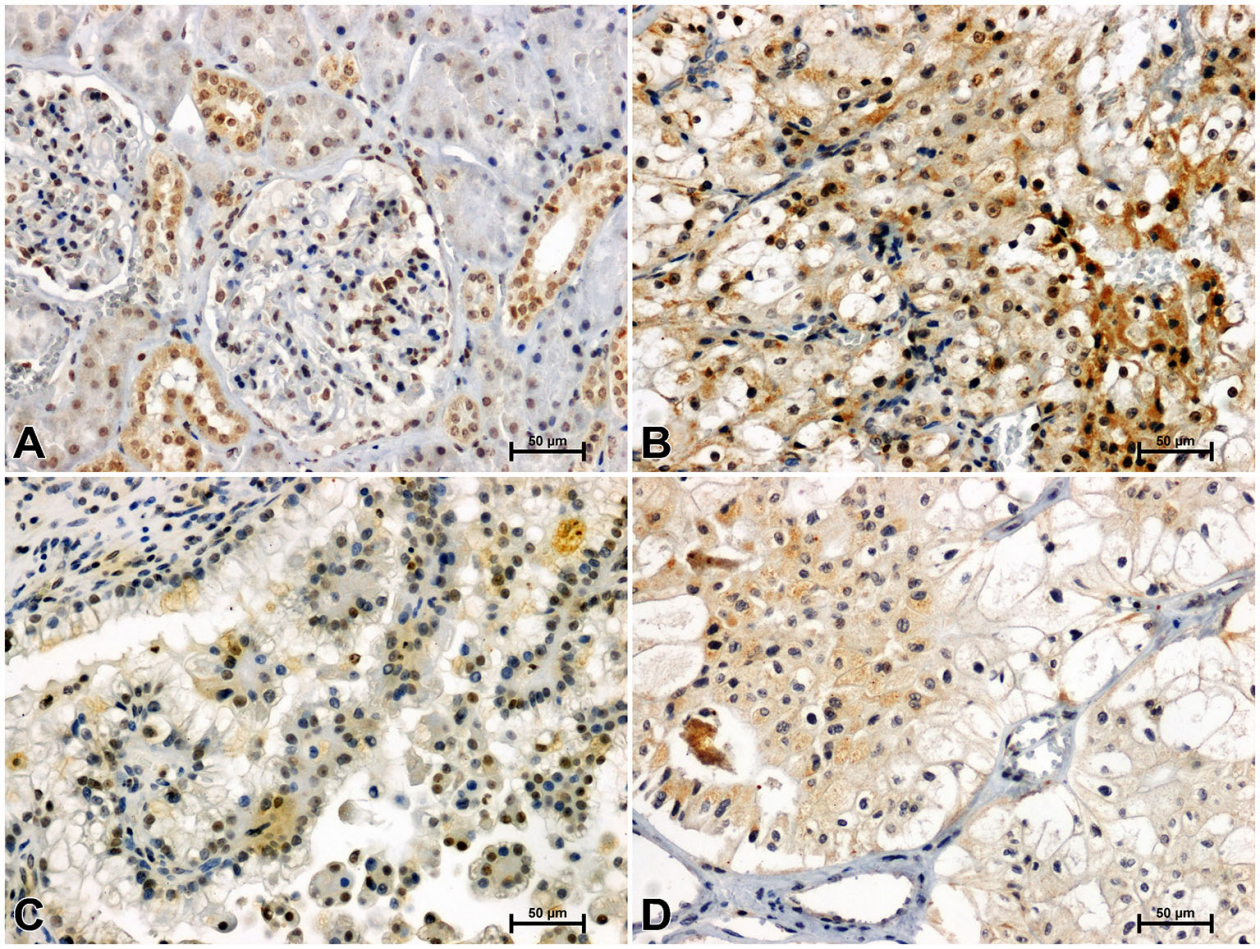
Immunohistochemical reaction determining CacyBP/SIP in non-malignant kidney **(A)** and clear cell RCC **(B)**, papillary RCC **(C)**, chromophobe RCC **(D)**.

**Figure 6:**
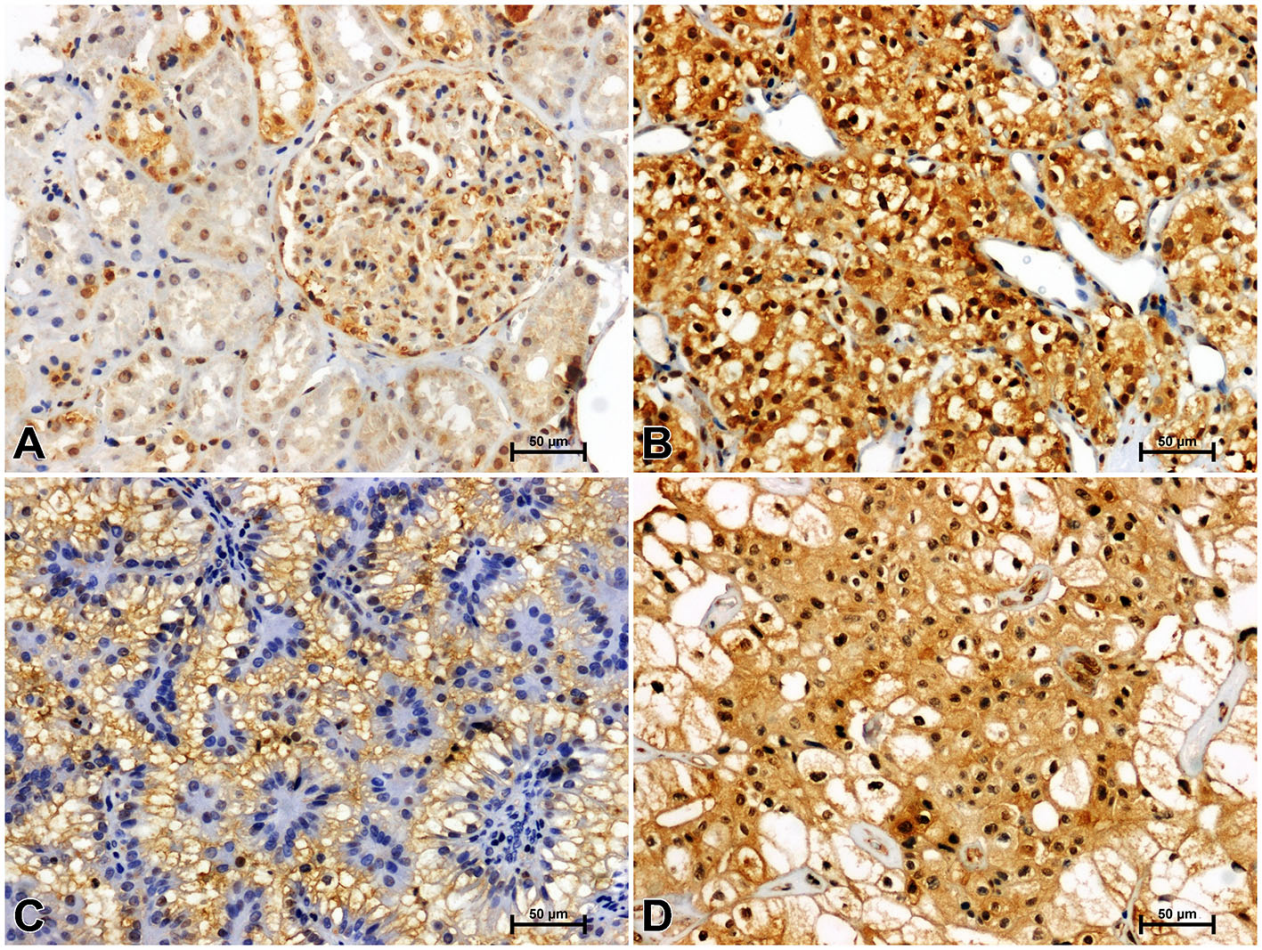
Representative immunohistochemical photomicrographs of LMP7 in non-malignant kidney **(A)** and clear cell RCC **(B)**, papillary RCC **(C)**, chromophobe RCC **(D)**.

In an unchanged kidney, there was a weak or moderate immune response against WNT10A in the renal tubules and very delicate WNT10A immunostaining in the glomeruli (**Figure 1A**). Considerably stronger immunoreactivity for WNT10A was found in clear cell RCC compared to non-malignant tissue (**Figure 1B**). In papillary RCC, WNT10A immunoreactivity was also increased compared to non-cancerous kidney, but to a lesser degree than in clear cell RCC (**Figure 1C**). There were no noticeable differences in the intensity of WNT10A immunostaining between chromophobe RCC and non-malignant tissue (**Figure 1D**).

Immunodetection of Fzd5 in non-malignant kidney resulted in medium or bright brown staining of the renal tubules and weak reactivity in the glomeruli (**Figure 2A**). The intensity of reaction against Fzd5 was significantly higher in RCC tissue (**Figures 2B–D**) compared to unchanged kidney. The highest immunoreactivity for Fzd5 was observed in clear cell RCC (**Figure 2B**), while the lowest increase in Fzd5 intensity was observed in chromophobe RCC in comparison to other histological types of RCC studied (**Figure 2D**).

Immunohistochemistry showed the presence of β-catenin in the renal tubules of non-cancerous kidney tissue. Distal tubules showed moderate β-catenin immunolabeling, while in proximal tubules the reaction against β-catenin was weak (**Figure 3A**). In clear cell RCC, significantly increased immunoreactivity of β-catenin was observed compared to non-cancerous control. The β-catenin immunosignal was very strong and located mainly in the peripheral area of malignant cells cytoplasm (**Figure 3B**). A significant increase in β-catenin immunoreaction was also observed in papillary RCC (**Figure 3C**), although the observed intensification of β-catenin reactivity was lower than in clear cell RCC. In chromophobe RCC, the intensity of β-catenin staining was also stronger compared to unchanged renal tissue, but the observed change in β-catenin immunoreactivity was least pronounced in comparison with other studied histological types of RCC (**Figure 4D**).

Antisera against GSK-3ß moderately immunostained renal tubules and glomeruli in non-malignant kidney (**Figure 4A**). GSK-3ß immunoreaction was noticeably increased in clear cell RCC (**Figure 4B**) compared to non-malignant kidney. In papillary RCC (**Figure 4C**) and chromophobe RCC (**Figure 4D**), intensity of GSK-3ß immunoreactivity was lower compared to clear cell RCC and did not differ from that observed in unchanged kidney.

CacyBP/SIP immunostaining in non-cancerous kidney revealed its location in the renal tubule system and glomeruli. In the distal tubules, moderate immunoreaction against CacyBP/SIP was observed, whereas in the proximal tubules and glomeruli, immunoreactivity for CacyBP/SIP was weak (**Figure 5A**). A much stronger intensity of the CacyBP/SIP immunosignal was observed in clear cell RCC, as compared to unchanged renal tissue (**Figure 5B**). Immunohistochemical reaction against CacyBP/SIP in papillary RCC (**Figure 5C**) and chromophobe RCC (**Figure 5D**) was weaker compared to clear cell RCC and was similar to that in non-cancerous control tissue.

Immunoidentification of LMP7 in non-cancerous kidney tissue gave a weak response in the proximal tubules and moderate reactivity in the distal tubules and glomeruli (**Figure 6A**). Greater immunoreactivity for LMP7 was noted in clear cell RCC compared to unchanged kidney (**Figure 6B**). Immunostaining of LMP7 in papillary RCC (**Figure 6C**) and chromophobe RCC (**Figure 6D**) was increased compared to non-malignant renal tissue, although it was lower than in clear cell RCC.

### Quantitative Analysis

The results of densimetric tests confirmed visually perceptible differences in the intensity of immunoreaction against WNT10A, Fzd5, β-catenin, GSK-3ß, CacyBP/SIP, and LMP7 between studied histological types of RCC and non-cancerous control samples (**Table 2**).

**Table 2:** The intensity of immunoreaction determining WNT10A, Fzd5, β-catenin, GSK-3ß, CacyBP/SIP, LMP7 in clear cell RCC, papillary RCC, chromophobe RCC, and non-malignant renal tissue (mean ± SE).

Group of patients	Intensity of immunohistochemical reaction in kidneyScale from 0 (white pixel) to 255 (black pixel)					
	WNT10A	Fzd5	β-catenin	GSK-3ß	CacyBP/SIP	LMP7
Control	76.0 ± 2.93	95.1 ± 2.01	101.4 ± 2.17	107.4 ± 2.17	86.2 ± 3.40	95.5 ± 154.9
Clear cell RCC	152.2 ± 4.62^a^ ^↑^	185.0 ± 2.60^a^ ^↑^	167.1 ± 2.71^a^ ^↑^	144.6 ± 2.90^a^ ^↑^	131.1 ± 3.58^a^ ^↑^	154.9 ± 3.04^a^ ^↑^
Papillary RCC	113.5 ± 5.00^a^ ^↑^ ^b^ ^↓^	154.9 ± 5.42^a^ ^↑^ ^b^ ^↓^	134.6 ± 3.39^a^ ^↑^ ^b^ ^↓^	110.5 ± 2.35^b^ ^↓^	79.7 ± 2.10^b^ ^↓^	114.4 ± 2.19^a^ ^↑^ ^b^ ^↓^
Chromophobe RCC	73.7 ± 2.30^c^ ^↓^ ^d^ ^↓^	121.1 ± 2.82^a^ ^↑^ ^c^ ^↓^ ^d^ ^↓^	119.8 ± 3.03^a^ ^↑^ ^c^ ^↓^ ^d^ ^↓^	107.1 ± 2.55^c^ ^↓^	79.7 ± 2.63^c^ ^↓^	114.3 ± 2.46^a^ ^↑^ ^c^ ^↓^

a p < 0.05—RCC vs control kidney.

b p < 0.05—papillary RCC vs clear cell RCC.

c p < 0.05—chromophobe RCC vs clear RCC.

d p < 0.05—chromophobe RCC vs papillary RCC.

↑—intensification of immunohistochemical reaction.

**↓**—weakening of immunohistochemical reaction.

### Real-Time PCR

**Figure 7:**
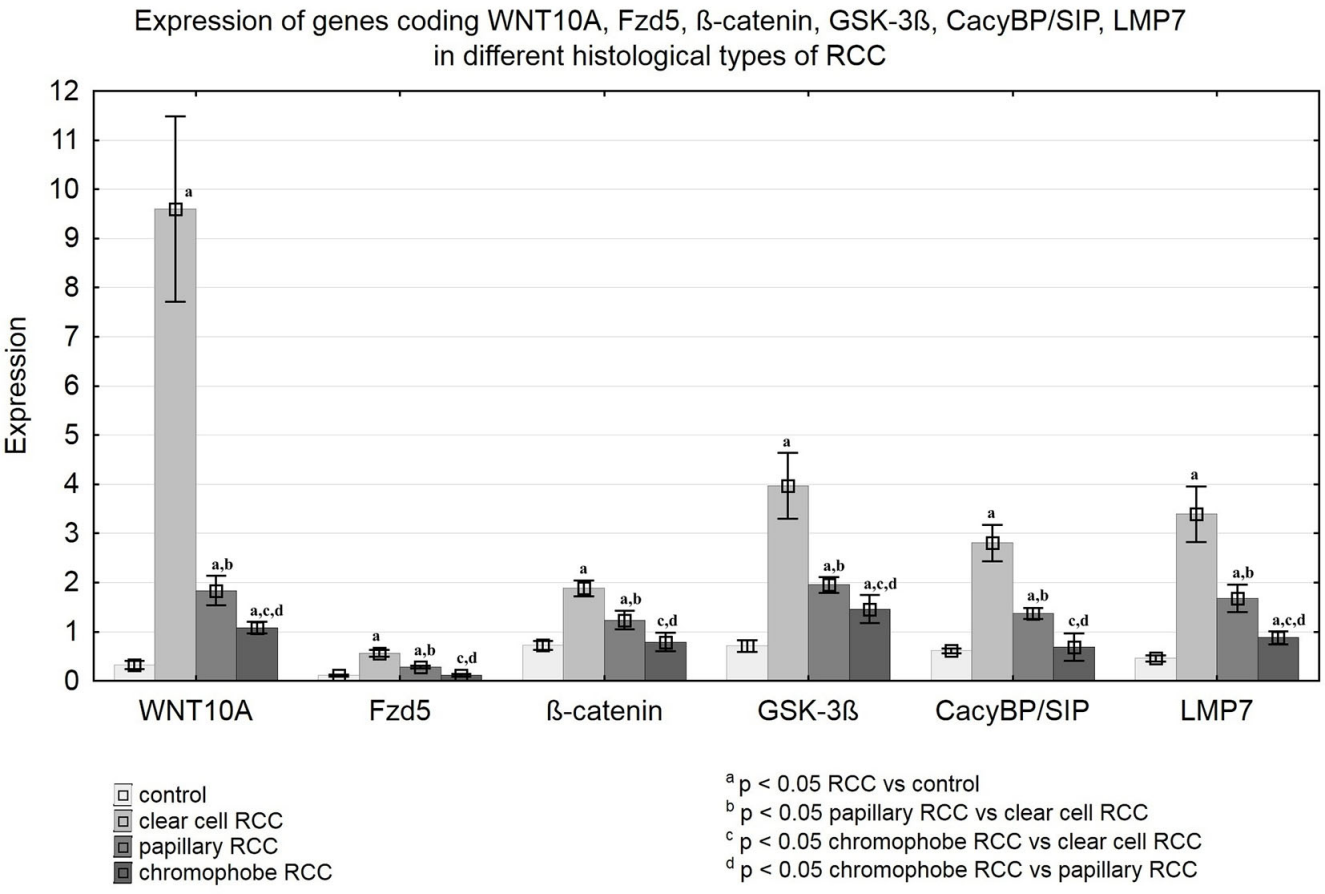
Expression of genes coding WNT10A, Fzd5, β-catenin, GSK-3ß, CacyBP/SIP, LMP7 in clear cell RCC, papillary RCC, chromophobe RCC and non-malignant renal tissue.

QRT-PCR analysis revealed a significant increase in the expression of the WNT10A, Fzd5, β-catenin, GSK-3ß, CacyBP/SIP, and LMP7 genes in clear cell RCC compared to non-malignant kidney. Similarly, noticeably increased expression of the tested genes was found in papillary RCC compared to unchanged renal tissue, but the observed increase was smaller than in clear cell RCC. The weakest expression of the analyzed genes was observed in chromophobe RCC compared to other studied histological types of RCC. Expression of WNT10A, GSK-3ß, and LMP7 in chromophobe RCC was marginally elevated compared to non-cancerous control, while expression of the Fzd5, β-catenin and CacyBP/SIP genes remained at the level observed in unchanged kidney (**Figure 7** and **Table 3**).

**Table 3:** The expression of genes coding WNT10A, Fzd5, β-catenin, GSK-3ß, CacyBP/SIP, LMP7 in clear cell RCC, papillary RCC, chromophobe RCC and non-malignant renal tissue (mean ± SE).

Group of Patients	The expression of genes coding WNT10A, Fzd5, β-catenin, GSK-3ß, CacyBP/SIP, LMP7 in kidney					
	WNT10A	Fzd5	β-catenin	GSK-3ß	CacyBP/SIP	LMP7
Control	0.32 ± 0.084	0.11 ± 0.019	0.72 ± 0.086	0.71 ± 0.117	0.61 ± 0.004	0.46 ± 0.060
Clear cell RCC	9.60 ± 1.889^a^ ^↑^	0.56 ± 0.069^a^ ^↑^	1.88 ± 0.163^a^ ^↑^	3.97 ± 0.666^a^ ^↑^	2.81 ± 0.374^a^ ^↑^	3.39 ± 0.562^a^ ^↑^
Papillary RCC	1.84 ± 0.300^a^ ^↑^ ^b^ ^↓^	0.28 ± 0.033^a^ ^↑^ ^b^ ^↓^	1.24 ± 0.189^a^ ^↑^ ^b^ ^↓^	1.95 ± 0.164^a^ ^↑^ ^b^ ^↓^	1.37 ± 0.111^a^ ^↑^ ^b^ ^↓^	1.68 ± 0.276^a^ ^↑^ ^b^ ^↓^
Chromophobe RCC	1.08 ± 0.115^a^ ^↑^ ^c^ ^↓^ ^d^ ^↓^	0.11 ± 0.001^c^ ^↓^ ^d^ ^↓^	0.79 ± 0.185^c^ ^↓^ ^d^ ^↓^	1.46 ± 0.280^a^ ^↑^ ^c^ ^↓^ ^d^ ^↓^	0.69 ± 0.275^c^ ^↓^ ^d^ ^↓^	0.88 ± 0.130^a^ ^↑^ ^c^ ^↓^ ^d^ ^↓^

a p < 0.05—RCC vs control kidney.

b p < 0.05—papillary RCC vs clear cell RCC.

c p < 0.05—chromophobe RCC vs clear RCC.

d p < 0.05—chromophobe RCC vs papillary RCC.

↑—increased expression of gene.

**↓**—decreased expression of gene.

## Discussion

According to the latest data from the International Agency for Research on Cancer, approximately 403,000 new cases of kidney cancer were diagnosed globally in 2018 (24). Kidney cancer is the ninth and fourteenth most common malignancy in men and women, respectively (25). Each year, between 150 and 175,000 patients die from this cancer, which ranks it third in terms of mortality among urological cancer (1, 24, 25). Advancing our knowledge of the mechanisms involved in RCC pathogenesis is necessary for improving the effectiveness of prevention and treatment of this malignant disorder.

Considering the important role of the Wnt/ß-catenin pathway in carcinogenesis and the close relationship between WNT/β-catenin signaling, CacyBP/SIP and proteasomal activity, it seemed worthwhile to undertake research aimed at comparing the WNT/β-catenin pathway, CacyBP/SIP and LMP7 immunoproteasome in human samples of different RCC histological types.

In presented study were examined 50 patients with various histological types of RCC: 35 with clear cell RCC, 12 with papillary RCC, 3 with chromophobe RCC. Men represented almost two-third of the studied RCC cases, which is consistent with literature data indicating on higher incidence of RCC in men than in women (1). Our study showed markedly increased immunoreactivity for WNT10A, Fzd5, β-catenin, GSK-3ß, CacyBP/SIP, and LMP7 in clear cell RCC compared to unchanged kidney tissue. In papillary RCC, immunoreactivity of the tested antibodies (except GSK-3ß and CacyBP/SIP) was also increased compared to non-malignant kidney, but it was weaker than in clear cell RCC. Reaction intensity for GSK-3ß and CacyBP/SIP in papillary RCC was comparable to that observed in non-malignant kidney. Immunoreactivity of the tested antibodies in chromophobe RCC was at the level observed in non-cancerous kidney cells and only for the Fzd5, β-catenin and LMP7 antibodies was it marginally stronger compared to control samples. Gene expression analysis confirmed the results obtained in immunohistochemical reactions.

Alterations in Wnt/ß-catenin pathway activity in RCC have been revealed in several previous investigations. Hsu et al. (11) demonstrated significantly increased expression of the WNT10A gene in RCC lines and in tissues of human RCC compared to non-cancerous control samples. In subsequent immunohistochemical studies, the authors also found increased content of WN10A and β-catenin in human RCC compared to normal renal tissue (11). Janssens et al. (12) revealed increased expression of some Fzd receptors in human RCC samples, including Fzd5 compared to a healthy kidney. Subsequent Western blot analysis confirmed the increase in Fzd5 content in kidney cancer compared to a healthy kidney (12). Liu et al. (9) demonstrated enhanced levels of β-catenin in the blood of patients with RCC in comparison to healthy individuals. Similarly, the authors noted increased expression of the β-catenin gene and elevated peptide content in RCC samples compared to non-malignant renal tissue (9). Other studies have shown elevated level of GSK-3ß in human RCC samples and cultured RCC cell lines, as compared to non-cancerous control samples (13). The reports mentioned above describe changes in WNT/β-catenin signaling only in clear cell RCC. To the best of our knowledge, the present study is the first comparative assessment of this signaling pathway in various histological types of RCC.

The proliferative potential of cancer cells reflects tumor aggressiveness and is strongly correlated with clinical prognosis. Mehdi et al. (26) examined proliferation rates in various histological types of RCC using Ki-67 and mini-chromosome maintenance 2 (MCM-2) markers. The study showed an increased level of proliferative markers in all samples of RCC, wherein in clear cell RCC the proliferation index was the most enhanced compared to papillary RCC and chromophobe RCC (26). Several *in vitro* studies have demonstrated that the WNT/β-catenin pathway plays an important role in regulating the proliferation of kidney cancer cells. Hsu et al. (11) stated that forced overexpression of WNT10A in RCC cell lines resulted in enhanced proliferation and invasion of malignant cells, while silencing WNT10A and the β-catenin gene leads to cell cycle arrest and reduced renal cancer cell migration. Yang et al. (27) genetically suppressed the expression of Fzd8 receptors in RCC cell lines and noticed lower proliferative potential in cancer cells with knocked-down Fzd8 gene compared to those without modification. Von Schulz-Hausmann et al. (28), Koller, et al. (29), and Sasamura et al. (30) demonstrated that incubation of renal cancer cell cultures with WNT/β-catenin signaling inhibitors slowed the proliferation of neoplastic cells. Bilim et al. (13), Kawazoe et al. (14) and Pal et al. (15) found that inactivation of the GSK-3ß gene or treatment with GSK-3ß inhibitors reduced the proliferation of cultured RCC cells. In subsequent studies, the scientists injected renal cancer cells into mice subcutaneously and investigated the effect of pharmacological inhibition of GSK-3ß on tumor growth. They noticed that in mice that received GSK-3ß inhibitors, the tumor grew slower than in untreated animals (13–15). Considering the above, greater proliferative potential of cancer cells in clear cell RCC compared to other RCC histological subtypes might be associated with the highest increase in the WNT/β-catenin pathway observed in clear cell RCC as compared to papillary RCC and chromophobe RCC.

WNT/β-catenin signaling is also implicated in controlling renal cancer cell survival. Activation of the WNT/β-catenin pathway inhibits cell apoptosis by reducing the release of cytochrome c from mitochondria and decreasing caspase levels (31). RCC cells with forced WNT10A overexpression have been shown to be more resistant to the cytotoxic effects of chemotherapy drugs (11). Conversely, suppression of WNT10A and β-catenin expression in RCC cells increases their susceptibility to chemotherapy (11). Silencing the Fzd8 receptor gene in cultured RCC cells significantly reduces their viability compared to cells with unmodified Fzd8 receptor expression (27). Similarly, treatment of RCC cells with WNT/β-catenin signaling inhibitors negatively affects their vitality (28–30). Genetic or pharmacological inhibition of GSK-3ß in RCC cells leads to cancer cell apoptosis, causing disturbances in intracellular energy homeostasis, downregulation of anti-apoptotic proteins and activation of caspases (13–15). Our research results indicate that in RCC, the WNT/β-catenin signaling pathway is activated with varying degrees of intensity depending on the type of cancer. Our findings may suggest differences in the regulation of signaling pathways determining the survival of malignant cells in examined histological subtypes of RCC.

Clinical data indicate far more frequent occurrence of metastases in clear cell RCC and papillary RCC in comparison to chromophobe RCC (3, 4). Neoplastic cells become invasive and spread from the primary site when they lose their intercellular connection and detach from surrounding cells (32). The basic components of cell-cell junctions are cadherins (32). Langner et al. (33), Kuehn et al. (34) and Shen et al. (35) evaluated immunohistochemically cadherin expression in human RCC tissues. These studies revealed positive immunostaining for cadherins only in a small percentage of clear cell RCC and papillary RCC samples, while in almost all cases of chromophobe RCC immunoreactivity for cadherins was observed (33–35). Gerharz et al. (36) described the ultrastructural aspects of cancer cells in various histological types of RCC and found that desmosomal junctions were rarely seen in clear cell RCC and papillary RCC, while in chromophobe RCC, typical desmosomes density was observed. The above findings indicate relatively undisturbed interactions between cells in chromophobe RCC, and significant disruption in the intercellular junction in clear cell RCC and papillary RCC. β-catenin plays a fundamental role in two different cellular processes: Wnt-mediated transcription activation and coordination of intercellular junctions by forming complexes with membrane cadherins (37). Proper balancing between the transcriptional and adhesive activity of β-catenin is crucial for maintaining cell function (37). In the state of chronic overactivation of the WNT/β-catenin pathway, β-catenin may be directed to transcriptional gene regulation at the expense of binding β-catenin to cadherin and its participation in intercellular adhesion. In view of the above, it might be suspected that, there are less severe disorders in intercellular junctions in chromophobe RCC compared to clear cell RCC and papillary RCC due to different intensity of changes in WNT/β-catenin signaling in the studied histological subtypes of RCC. Hence, different degrees of alteration in WNT/β-catenin signaling between clear cell RCC, papillary RCC and chromophobe RCC revealed in our study can provide a possible explanation for the distinct invasive behavior of the examined histological types of RCC.

The histological RCC subtype is an important clinical prognostic factor. In patients with clear cell RCC, prognosis is worse than in patients with papillary RCC and chromophobe RCC. The 5-year survival rate in clear cell RCC is 43% to 89%, while in papillary RCC and chromophobe RCC, it is 57% to 86% and 76% to 100%, respectively (3, 4). Some recent evidence has demonstrated a significant relationship between WNT/β-catenin signaling and the survival of patients with RCC. It has been revealed that higher serum β-catenin levels and increased content of WNT10 and β-catenin in cancer tissue are associated with higher mortality due to RCC (9, 11). It may be hypothesized that greater activation of the WNT/β-catenin pathway in clear cell RCC in comparison to papillary RCC and chromophobe RCC, as stated in the present report, might be the mechanism responsible for a worse prognosis in clear cell RCC compared to other examined histological subtypes of RCC.

Recent literature data indicates abnormal levels of CacyBP/SIP in various types of cancer such as gastric cancer, breast cancer, pancreatic cancer, colorectal cancer and brain cancer (16–18). However, there is only one report in the available literature on changes in CacyBP/SIP in renal cancer and therefore the role of this peptide in RCC cancerogenesis is not fully understood (19). Sun et al. (19) showed decreased CacyBP/SIP immunoreactivity and reduced CacyBP/SIP gene expression in human RCC samples and RCC cell lines compared to non-cancerous control tissue samples. On the contrary, our study revealed an intensified CacyBP/SIP immunostaining and increased CacyBP/SIP gene expression in human RCC tissue compared to non-cancerous kidney. This discrepancy may be due to differences in the methods used. The role of CacyBP/SIP in cancer progression appears to be contradictory – it has been shown to either promote or inhibit the proliferation and invasiveness of cancer cells, depending on the type of cancer (16–19). Investigation by Sun et al. (19) demonstrated, that injecting mice with CacyBP/SIP overexpressing RCC cells reduces their proliferative potential and carcinogenicity. Similar tumor-suppressory effect of CacyBP/SIP was stated in studies on gastric cancer, breast cancer and astrocytoma. On the other hand oncogene role of CacyBP/SIP was demonstrated in pancreas cancer and glioma. In colorectal cancer, CacyBP/SIP has been shown to reduce proliferation while enhancing the invasion and migration of cancer cells (16, 18). Considering current incomplete state of knowledge about role of CacyBP/SIP in RCC cancerogenesis, more research needs to be done to determine precisely the consequences of CacyBP/SIP alterations in RCC cancer.

Recent investigations have shown that in RCC cancer interferes the presentation of antigens on the cell surface to avoid being recognized and destroyed by the immune system (20–22). The antigen presentation process involves immunoproteasomes that produce antigenic peptides and MHC I complexes that exhibit surface antigens for immunologically competent cells (20–22). Seliger et al. (20, 21) and Atkins et al. (22) demonstrated significant downregulation of MHC complexes in human RCC. The authors also demonstrated weakened or negative LMP7 immunosignaling in a large proportion of human RCC samples studied, revealing immunoproteasomes expression deficiency in RCC cancer (20–22). It has been proposed that observed abnormalities in the expression of MHC I and LMP7 in RCC tissue contribute to the suppression of tumor antigen presentation and may be a mechanism of immune escape in RCC cancer (20–22).

In contrast to the findings presented above, our report showed stronger immunostaining for LMP7 and increased expression of the LMP7 gene in RCC human tissue compared to a healthy kidney. Malignant cells have uncontrolled proliferative potential and enhanced metabolism, and therefore are more likely to accumulate defective proteins (38). Therefore, accelerated proteasomal activity has been found in many types of cancer in response to increased demand for protein turnover (38). Perhaps the greater immunoreactivity of LMP7 and increased expression of the LMP7 gene in RCC tissue observed in our study, compared to unchanged kidney tissue, may be associated with increased proteasomal processing of proteins in malignant cells.

In summary, our report shows significant alterations in the level and expression of the WNT/β-catenin pathway, CacyBP/SIP and LMP7 genes in human RCC tissues, which were dependent on the histological type of the tumor. The results of the study suggest an important role of the WNT/β-catenin pathway, CacyBP/SIP and LMP7 in RCC carcinogenesis and may indicate new aspects of pathomechanisms leading to differences in the biology of clear cell, papillary, and chromophobe RCC.

## Data Availability Statement

The raw data supporting the conclusions of this article will be made available by the authors, without undue reservation.

## Ethics Statement

The studies involving human participants were reviewed and approved by Bioethics Committee, Medical University of Bialystok (R-I-002/282/2019). The patients/participants provided their written informed consent to participate in this study.

## Author Contributions

IK and ZP: conceived of and designed the experiments. ZP, MA, MN, and GM: analyzed the data. IK, MA, MN, and GM: contributed reagents/materials/analysis tools. Writing—original draft preparation: ŻP. Writing—review and editing: IK. All authors contributed to the article and approved the submitted version.

## Funding

This work was supported by statutory funds from the Medical University of Bialystok.

## Conflict of Interest

The authors declare that the research was conducted in the absence of any commercial or financial relationships that could be construed as a potential conflict of interest.
